# Phases in fine volcanic ash

**DOI:** 10.1038/s41598-023-41412-x

**Published:** 2023-09-21

**Authors:** Adrian Hornby, Esteban Gazel, Claire Bush, Kyle Dayton, Natalie Mahowald

**Affiliations:** Earth and Atmospheric Sciences, Cornell University, Ithaca, NY USA

**Keywords:** Mineralogy, Volcanology

## Abstract

Volcanic ash emissions impact atmospheric processes, depositional ecosystems, human health, and global climate. These effects are sensitive to the size and composition of the ash; however, datasets describing the constituent phases over size ranges relevant for atmospheric transport and widely distributed impacts are practically nonexistent. Here, we present results of X-ray diffraction measurements on size-separated fractions of 40 ash samples from VEI 2–6 eruptions. We characterize changes in phase fractions with grainsize, tectonic setting, and whole-rock SiO_2_. For grainsizes < 45 μm, average fractions of crystalline silica and surface salts increased while glass and iron oxides decreased with respect to the bulk sample. Samples from arc and intraplate settings are distinguished by feldspar and clinopyroxene fractions (determined by different crystallization sequences) which, together with glass, comprise 80–100% of most samples. We provide a dataset to approximate glass-free proportions of major crystalline phases; however, glass fractions are highly variable. To tackle this, we describe regressions between glass and major crystal phase fractions that help constrain the major phase proportions in volcanic ash with limited a priori information. Using our dataset, we find that pore-free ash density is well-estimated as a function of the clinopyroxene + Fe-oxide fraction, with median values of 2.67 ± 0.01 and 2.85 ± 0.03 g/cm^3^ for intraplate and arc samples, respectively. Finally, we discuss effects including atmospheric transport and alteration on modal composition and contextualize our proximal airfall ash samples with volcanic ash cloud properties. Our study helps constrain the atmospheric and environmental budget of the phases in fine volcanic ash and their effect on ash density, integral to refine our understanding of the impact of explosive volcanism on the Earth system from single eruptions to global modeling.

## Introduction

Following explosive volcanic eruptions, the transport and deposition of volcanic ash result in widespread and diverse impacts in the environment^1–3^. Even a few millimeters of ashfall can cause cascading impacts^4, 5^ that result in myriad societal, ecological, and health issues^6–8^. Airborne volcanic ash can also acutely impact atmospheric processes and radiative forcing^9–12^ and present hazards to the global aviation industry^13^. The size, chemistry, and constituent phases of volcanic ash play a primary role on its impact in receiving environments^2, 14, 15^; constraining these properties also helps to infer radiative properties that improve the capacity to detect ash clouds, forecast their dispersion and impact and quantify airborne ash concentration^16–18^. The finer the ash, the greater the potential for dispersion, remobilization^19^, transport and atmospheric lifetime^20^, and the greater the environmental reactivity^21, 22^, including hazards to respiratory health^23^. Finally, a better understanding of iron- and phosphorus-bearing phase fractionation and distribution is important for land and ocean biogeochemistry^24, 25^.

Despite the critical impact of volcanic ash on the Earth system following a large eruption (potentially on a global scale), constraining the modal composition of volcanic ash remains a challenge and it is often misrepresented or oversimplified. Unfortunately, this is particularly true for the fine volcanic ash capable of prolonged atmospheric transport, which has been neglected in preference of better constrained inputs of other aerosols in atmospheric science^26^ and in preference of coarser fallout particles in volcanology^27^. Due to terminology differences between these research fields, a unique definition of fine volcanic ash is missing; fine (< 2.5 μm) and coarse aerosols (2.5-10 μm) in atmospheric science versus the coarser sedimentological criteria from ash fallout traditionally studied in volcanology; it might be argued that both definitions are poorly suited to volcanic ash clouds, where eruption columns force particles with a broad size distribution into the atmosphere. A definition for ‘very fine ash’ at < 30 μm based on fluid-dynamic settling behavior^28^ may be a good compromise. Here, we examine ash in two sieved size ranges < 25 µm and 25-45 µm diameter, a reasonable approximation of the median size ranges found in medial-to-distal tephra deposits (e.g., Table 1 in ref.^27^), representing a step towards understanding volcanic ash particles at sizes comparable to those considered by atmospheric scientists.

**Figure 1:**
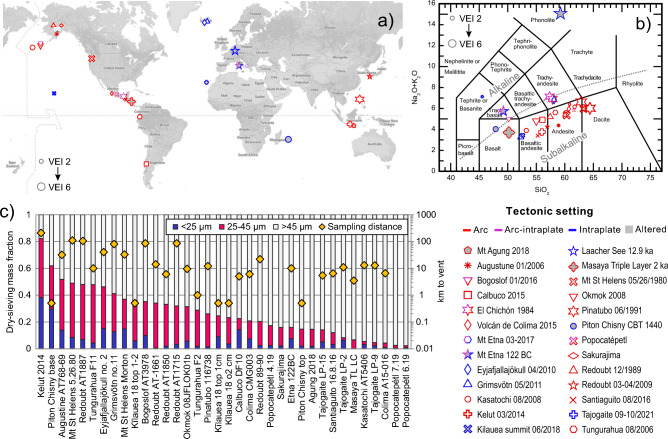
Global eruption locations and bulk chemistry. (**a**) Location of the volcanoes that produced volcanic ash used in this study (further details in Supplementary Table 1) The symbol size is scaled to the eruption volcanic explosivity index (VEI). (**b**) Total alkalis to silica (TAS) diagram^34^ showing bulk chemistry in wt% oxides retrieved from the literature for the studied samples. Compiled data and references are provided in Supplementary Tables 2 and 7. Multiple data points bracket the range of chemistry for an eruption where the erupted materials were heterogenous. All symbols and colors in panels a-b follow the legend. Bicolored symbols indicate more complex arc-intraplate tectonic settings, with the bottom-right color indicating the dominant tectonic setting to which they were assigned for calculations and linear regressions. (**c**) Dry-sieving fractions for the grain size ranges used in the study are shown as stacked bars in order of total < 45 µm fraction. Gold diamonds show sampling distance from the eruptive vent. Map data © 2023 Google.

Existing datasets for modal composition are uncommon in the literature^14^ and specific comparisons between different magma-tectonic settings, eruption styles, and size fractions^29–31^ are very limited. Improving the characterization of the modal composition of fine volcanic ash is a high priority for a diversity of scientific fields and interdisciplinary research due to its widespread distribution following explosive eruptions. One common method to approximate the modal composition of volcanic materials is to calculate a ﻿‘normative’﻿ mineralogy by assigning all chemical components from a given bulk chemistry to ideal mineral phases following experimentally-derived phase relations^32^. The calculation rests on assumptions of equilibrium and anhydrous conditions, and complete crystallization of the magma and is often visualized as a highly simplified mineral proportion chart^33^. While this normative phase assemblage based on chemistry is useful for rock classification and provides a general idea of the likely components, the relevance for volcanic ash is limited; the underlying assumptions of full crystallization under equilibrium conditions are commonly interrupted by volatile-driven magma ascent and eruption, leading to quenching of remaining silicate melt to glass. As a result, classification based on chemical composition alone has become the standard for volcanic rocks, like the widely used binary diagram ‘total-alkalis-to-silica’ (TAS, Fig. 1). Unfortunately, neither the normative mineral modes nor the magma chemistry alone provide a quantitative basis for estimating the fractions of minerals and glass in an erupted magma.

Here, we present results of the first global characterization of the modal composition for ash samples at different grain sizes from a wide range of eruptions with differing magma-tectonic setting and magma chemistry. At the highest level, we categorize samples based on tectonic setting, broadly divided between subduction-related melting of the upper mantle resulting in volcanic arcs and active mantle upwelling in an intraplate setting. In volcanic arcs, primary melts are formed either via devolatilization of subducting oceanic crust and sediments leading to partial melting of the mantle wedge^35–37^ or via partial melting of the sediments or slab itself^38–40^, forming chains of arc volcanoes that track oceanic plate margins. For active upwelling in intraplate settings, primary melt is inferred to form in the mantle from a deeply rooted boundary layer (e.g., the core-mantle boundary^41, 42^ or the mantle’s transition zone^43, 44^) and ascends through the mantle in long-lived plumes that generate intraplate volcanism, such as at Hawaii. The characteristic erupted products of mantle plumes are ocean island and intraplate basalts; however, these upwellings may intersect with melts generated at plate boundaries leading to mixed-source magmatism^45, 46^. Arc magmas usually follow calc-alkaline (or rarely tholeiitic) crystallization sequences, while intraplate magmas are relatively reduced to moderately oxidized and evolve through either tholeiitic or alkaline (silica undersaturated) magma series^47, 48^. Thus, the contrasting origin of primary melts create discrepant physicochemical properties, which determine the crystallization trends that ultimately produce the diversity of eruptible magmas^49, 50^.

Volcanic ash forms from magma fragmentation and is a complex multiphase material with heterogeneous material properties within single grains and between different ash grains. This inherent variability represents, initially, the variability (and nonlinearity) present in eruptible magmas. However, due to the varying physical properties of crystalline phases and glass^29, 51^, size-dependent partitioning of ash mineral and glass content is expected during eruptive fragmentation and atmospheric transport processes. The properties of an airborne ash cloud and deposited (fallout) material also vary in space and time during atmospheric transport^52, 53^ due to settling controlled by the aerodynamic properties of single ash particles^54^ or particle aggregates^55^. Very few measurements of fine volcanic ash within volcanic ash clouds have been made and the evolution of ash cloud composition and physical or optical properties is poorly constrained.

Due to the uncertainty in volcanic ash modal phase compositions, our approach is to investigate and compare a broad dataset of natural samples of fine volcanic ash. It is important to state that the observed characteristics of volcanic ash are limited to samples from a specific eruption(s) and cannot a priori be considered a proxy for the ash characteristics of any volcanic system in subsequent or future activity. In addition, the properties of fine ash fallout and airborne volcanic ash are expected to differ; here we examine differences between fine and bulk ash samples at proximal-to-medial (< 100 km) distances from the eruptive vent (Fig. 1c). Our goal is to improve and facilitate estimations of the physical, chemical, and optical properties of fine volcanic ash following volcanic eruptions and their subsequent impacts on the Earth system.

## Results

**Figure 2:**
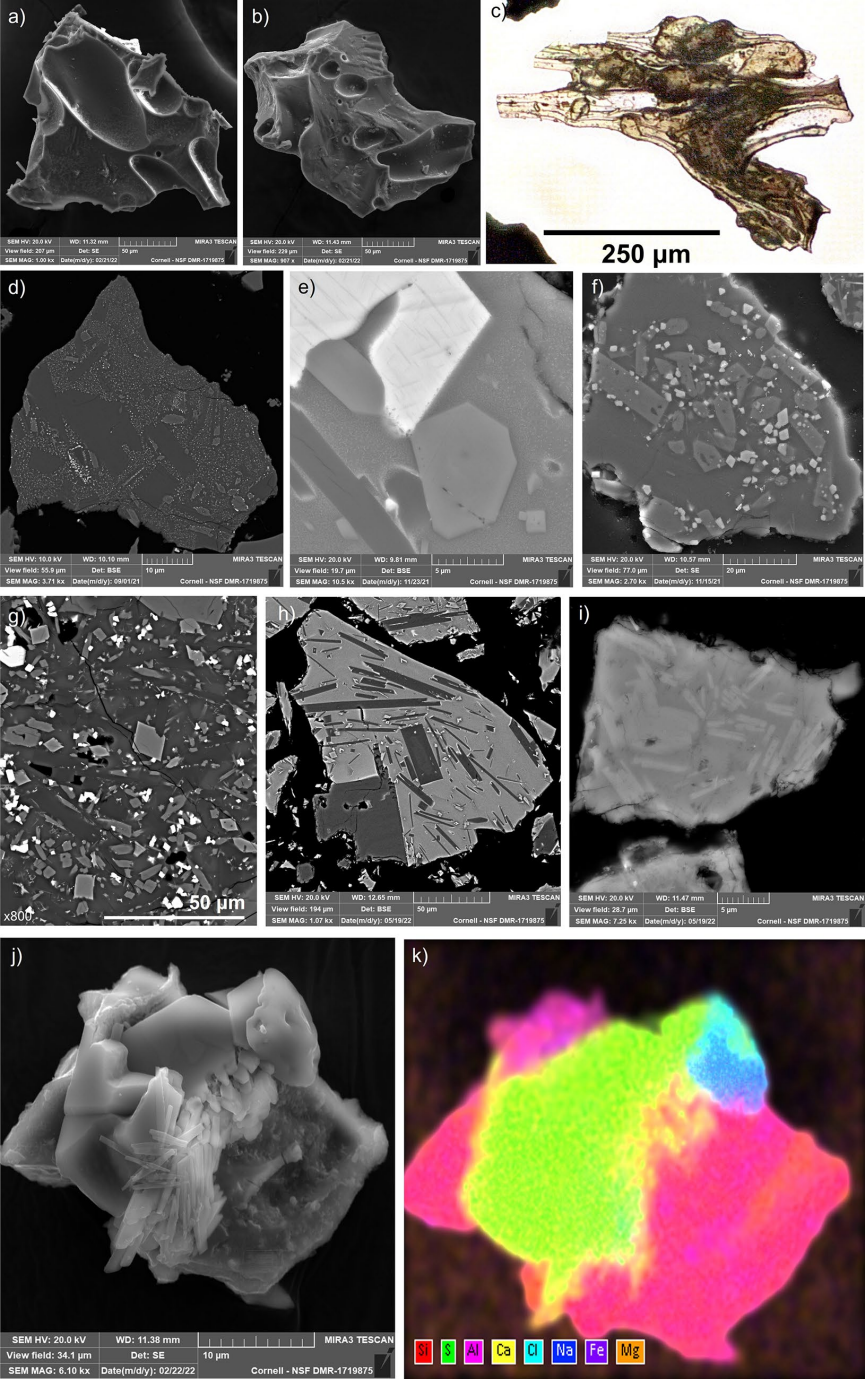
Typical morphology and phases of volcanic ash. (**a**–**b**) SEM secondary electron (SE) images showing typical forms of glassy volcanic ash with pore indentations from the Tajogaite 2021 eruption. (**c**) Microphotograph under plane-polarized light showing volcanic glass shard. (**d**) Backscattered electron (BSE) image showing intermediate arc sample from Tungurahua, with phenocrysts of plagioclase feldspar (dark) microlites of clinopyroxene (intermediate) and nanolites (specks) of Fe–Ti oxides. Glass is indistinguishable in greyscale from plagioclase, a common property of intermediate arc magmas. (**e**) BSE image showing Fe–Ti oxide phenocryst (bright), clinopyroxene (intermediate) and plagioclase (dark) microlites in a nanolite-rich glass (grey shade between plagioclase and pyroxene). (**f**) Crystal clusters are common, such as this set of clinopyroxene microlites and microphenocrysts associated with Fe–Ti oxides microlites in a BSE image from Tajogaite (2021). (**g**) BSE image of highly crystalline groundmass of Mt Etna 2017 sample, showing plagioclase, pyroxene and Fe-oxide microlites. Nanolite-rich glass can be seen as diffuse intermediate grey zones. (**h**) Ash grain from Okmok 2008 eruption, showing plagioclase phenocrysts and elongate microlites within an Fe-rich glass. (**i**) Sample from the Pinatubo 1991 eruption, showing plagioclase microlites in a Fe-poor felsic glass. (**j**) SE image from the Tajogaite 2021 eruption, showing salt crystals grown on an ash grain. (**k**) The identity of the salt crystals is seen by energy-dispersive spectroscopy (EDS), with a Ca-sulfate crystal in yellow and green, and halite in blues. All images produced by the authors using TESCAN Mira3, Bruker Esprit and Leica LAS-X software.

The ash samples used in this study possess a range of physical and chemical properties too diverse to list in this study, however detailed examination of many of the samples has been made and the references for some of these are included in Supplementary Table 1. Examples of the broad morphological, petrographic, and chemical features discussed are illustrated in Fig. 2. Most atmospheric ash consists of glass and different crystalline phases derived from fragmentation of magma, referred to as juvenile components. Fragmentation of parts of the volcanic conduit and edifice can cause ‘lithic fragments’ to become mixed with juvenile ash. The shape of ash particles is determined by the pre-eruptive magma properties, especially pore number density as well as pore and crystal fraction, and modulated by syn-eruptive deformation and fragmentation. Crystalline ash shape depends on the broken mineral phases. Initially, silicate melts may flow and stretch while ascending towards Earth’s surface, forming elongate bubbles and fluidal textures. Crystal content, size and shape also affect the texture and morphology of glassy particles. During eruption, magma is fragmented, forming angular and blocky fragments (Fig. 2a–c). Depending on eruption conditions, rapid cooling causes the silicate melt to quench to glass. Larger crystals can form single particles, freed from the magma during fragmentation. Smaller crystals, such as the microcrysts and nanolites in Fig. 2d–g, affect the magma mechanical strength and fracture behavior of the magma. Magmas erupting with high crystal fractions typically form blocky and low-porosity ash particles. Glass compositions can vary widely between different ash samples, as illustrated by the inversion in relative backscatter intensity compared to feldspar crystals in Fig. 2h and i. Variations in glass chemistry may also be measured in single particles due to the interaction of magmas of different chemistry prior to eruptions, which leads to mixing and mingling textures in volcanic glass^56^. Fresh ash particles commonly become mantled by secondary salts and brines^15, 57^, which can substantially change the surface properties, aggregation potential and environmental impacts of the particles (Fig. 2j–k).

**Figure 3:**
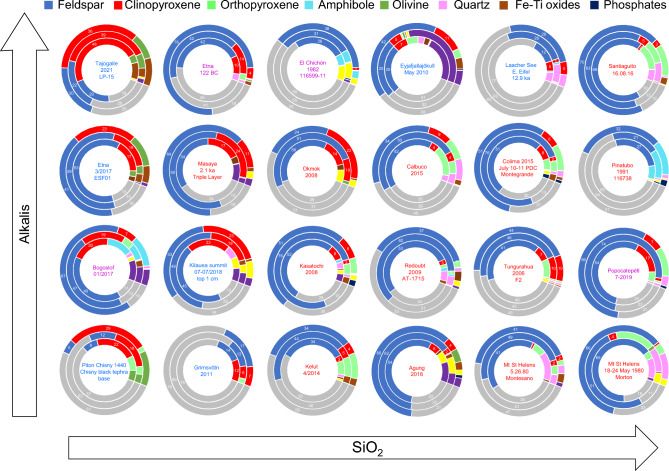
Phase fractions are represented as donuts, where the outer rings show bulk modal compositions, the central rings show the 25-45 μm fraction and the inner rings the < 25 μm fraction. Donuts are arranged so SiO_2_ increases to the right and alkalis increase upwards. A single sample was chosen where multiple eruptions from the same volcano were sampled, except for Mount St. Helens, where a sample from the initial blast deposit is compared to later magmatic eruption deposits. Major phases are labelled with the weight fraction of the phase to the nearest integer.

**Figure 4:**
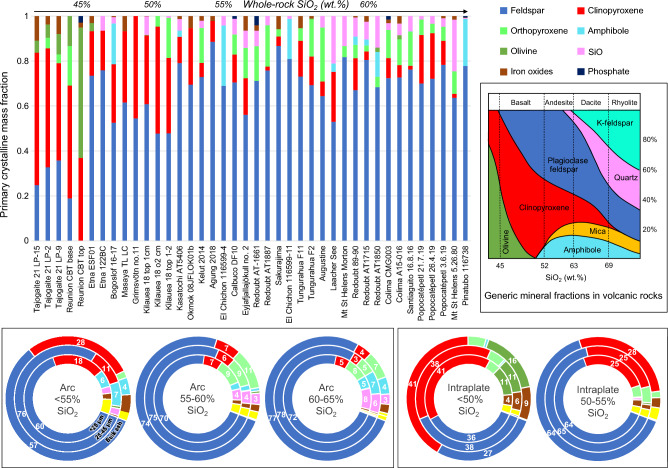
Primary mineral phases in fresh volcanic ash samples arranged by SiO2 content from left-to-right. Salts, ‘Other’ phases and glass have been removed from the plot and the remaining phases normalized to 100%. The plots here may be compared to the generic mineral abundance plots based on crystallization series that are commonly used in teaching and literature (an example is shown as an inset on the right of the figure, modified from images in the public domain, e.g., https://commons.wikimedia.org/wiki/File%3AMineralogy_igneous_rocks_EN.svg). Donut plots show average glass-free phase fractions in binned whole rock SiO_2_ fractions for arc samples (left) and intraplate samples (right). Different size fractions are represented by nested rings, as labeled in the left-hand donut. Numbers indicate the primary mineral fraction normalized on glass- and secondary-mineral-free basis. Note: different whole-rock SiO_2_ bins were used for arc and intraplate samples due to data distribution.

Using X-ray diffraction, we measured the composition of 40 size-separated samples from 23 volcanoes. We present the data for 24 samples including all source volcanoes in donut charts in Fig. 3, arranged approximately by whole-rock silica and alkali oxide weight fractions. The full dataset is provided in Supplementary Table 4. A few key observations stand out in this figure: first, the increase in clinopyroxene toward the low-silica samples; second, the ubiquitous dominance of feldspar and glass; third, the significant fractions of ‘other’ minerals in the Eyjafjallajökull, Agung, Bogoslof, K﻿īlauea and Masaya samples; fourth, the common (yet not ubiquitous) trend for lower glass in the < 25 µm (inner ring) fraction. A trend that is pronounced for some eruptions (for example in Kelut, Okmok, and Popocatépetl samples) is a peak in feldspar fraction in the 25-45 μm fraction. We also present the full dataset for primary minerals only (e.g., without volcanic glass, secondary salts or alteration minerals) in the < 25 µm fraction in Fig. 4, arranged by increasing silica fraction from right-to-left. We include the glass-free mineral fraction figure based on crystallization sequences commonly found in teaching and literature materials in the inset for comparison. Here, increases in clinopyroxene with corresponding decreases in plagioclase content can be clearly seen from <  ~ 50 ﻿wt% SiO_2_, together with a transition from quartz- to olivine-bearing ash < 45 wt% silica. These thresholds are considerably lower than in the idealized inset diagram; it is also apparent that plagioclase fractions tend to be higher and clinopyroxene lower than in our measurements than in the inset diagram, and that amphibole fraction can be significant but is only intermittently present and does not show a trend with silica. As a comparison, we present average phase proportions grouped by whole-rock SiO_2_ in five wt% bins for arc and intraplate settings. For arc samples with < 55 wt% SiO_2_, compositions contain less plagioclase, more clinopyroxene and less orthopyroxene than for samples with > 55 wt% silica. Intraplate ash samples show mineralogical changes < 50 wt% SiO_2_, (notably increased clinopyroxene, olivine and Fe-oxides and decreased plagioclase) from samples with 50–55 wt% SiO_2_. We note that in the average compositions, the 25–45 µm fraction bears more plagioclase than the bulk or the < 25 µm fractions in all cases, for arc and intraplate samples, but is very pronounced for the < 55 wt% silica arc samples.

**Figure 5:**
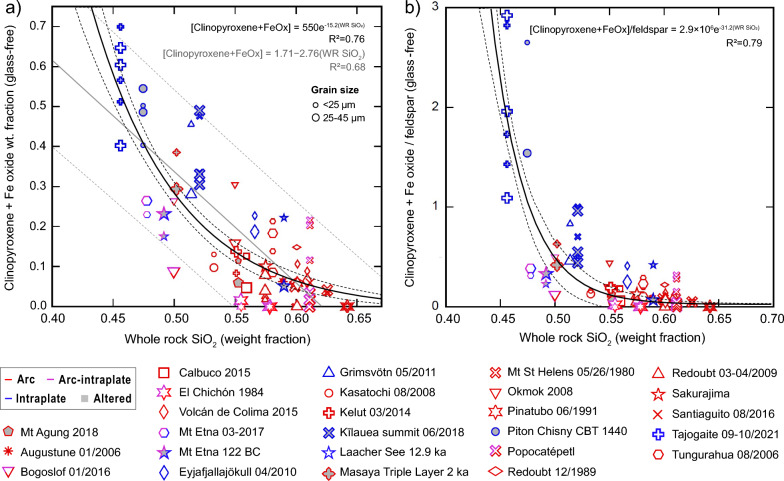
Scatter plots showing relationships between whole-rock SiO_2_ and primary mineral phase fractions (labeled ‘glass-free’). (**a**) Clinopyroxene + Fe oxides, normalized on a glass-free basis for < 25 and 25–45 µm fractions are fitted with an exponential regression (black line) and linear regression (grey line) to bulk silica with altered samples (symbols with grey fill) excluded. The grey dashed lines show the 95% confidence curves for individual data points; black dashed lines show 95% confidence for the exponential regression. (**b**) Identical to panel (**a**), but with the y-axis divided by feldspar fraction and no linear regression shown. Dashed lines shoe 95% confidence limits for individual data points. Symbol size indicates sieve fraction (smallest =  < 25 µm, largest = bulk (unsieved). Equations for regressions with the coefficient of determination (R^2^) are shown.

We further investigate the relationships between the primary crystal proportions by plotting the major phases for individual sieved samples against whole-rock SiO_2_ in Fig. 5a–b. We plot a linear regression describing decreasing clinopyroxene + Fe-oxide fraction with increasing SiO_2_ in Fig. 5a,

1 $$\left[ {{clinopyroxene} + {Fe\text{ }oxide}} \right] = 1.71 - 2.76 \cdot \left( {{\text{WR }} {\text{SiO}}_{2} } \right),$$

where WR SiO_2_ is whole rock silica weight fraction. An exponential fit is slightly better, with a coefficient of determination (R^2^) of 0.76 vs. 0.68, and appears to better model the arc data points:

2 $$\left[ {{clinopyroxene} + {Fe\text{ }oxide}} \right] = 550e^{{ - 0.14 \cdot \left( {{\text{WR }} {\text{SiO}}_{2} } \right)}} .$$

We also show that the ratio of clinopyroxene + Fe-oxide:feldspar can be described by an exponential linear regression

3 $$\frac{{\left[ {{clinopyroxene} + {Fe\text{ }oxide}} \right]}}{{\left[ {feldspar} \right]}} = 2.9 \cdot 10^{6} e^{{ - 31.2 \cdot \left( {{\text{WR }} {\text{SiO}}_{2} } \right)}} ,$$

with an R^2^ of 0.79.

Equations (1–3) allow the major crystalline phase proportions to be roughly estimated based on measured or estimated whole-rock SiO_2_ fraction alone, bearing in mind a considerable degree of error (e.g., 95% confidence limits for the linear regression through clinopyroxene + Fe-oxide data points are ± 20 wt%).

**Figure 6:**
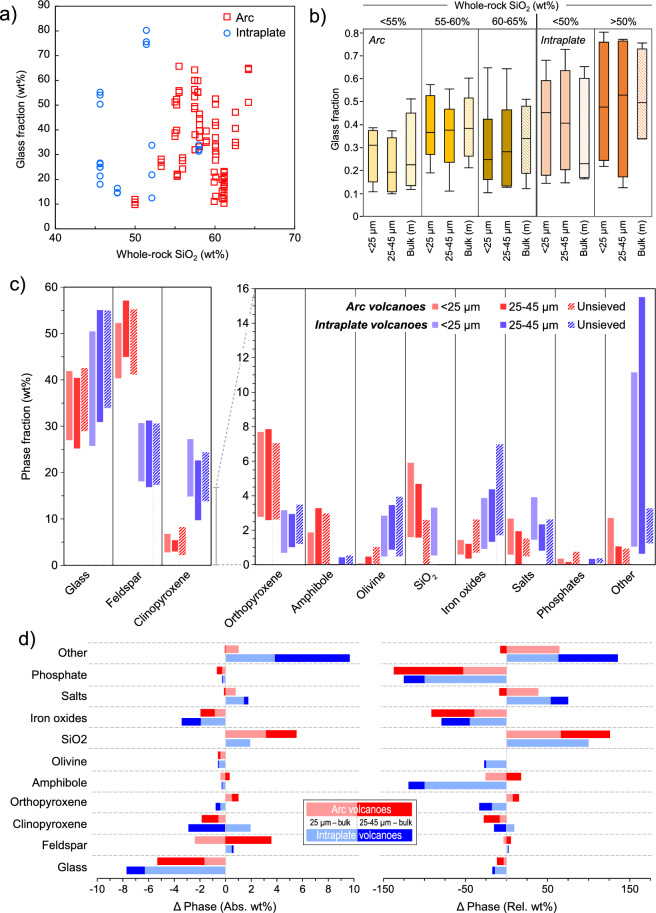
Average phase composition and sample standard error of the mean for arc and intraplate volcanic ash samples in the study. (**a**) Glass fraction is plotted against whole-rock SiO_2_ for all samples, showing poor correlation and wide range. (**b**) Box plots showing glass content binned by whole-rock SiO_2_ content and grain size. Arc samples are shown in yellow bars, intraplate samples in orange. (**c**) Sample standard deviation is shown as a bar for each phase for arc and intraplate ash samples. The y-axis scale is magnified for phases shown to the right of clinopyroxene to improve clarity of the minor phases. (**d**) Variations in phase abundance from the bulk for fine-sieved particles. Absolute variations (left) and relative variations (right) in weight fraction are plotted as stacked bars for each phase. Lighter colors represent the finest size range (< 25 μm) and darker colors the 25-45 μm range.

To this point, we have not included volcanic glass, a ubiquitous phase in juvenile volcanic materials but forming via quenching of remaining silicate melt in magma during an eruption. Glass fractions are highly variable and are poorly sensitive to whole-rock chemistry. No clear pattern is found either with data for < 25 μm ash samples (Fig. 6a) or for samples binned by WR SiO_2_ (Fig. 6b). Prior to calculating any average values, we reduced sampling bias by computing the mean for each phase in multiple samples from the same eruption. We also did not include highly altered samples in the calculation (symbols with gray shading in Fig. 1) except for the top 1 cm sample from the 2018 K﻿īlauea summit caldera, which had the least alteration of the K﻿īlauea samples. As expected, we found significant variations between arc and intraplate eruptions, particularly higher fractions of glass and clinopyroxene, and lower fractions of feldspar in the intraplate samples.

Patterns and variation in phase fractions with grainsize are highlighted in Fig. 5c, showing standard deviation bars for each phase. Standard deviation and standard error is higher for intraplate volcanoes due to lower sample numbers. Within our dataset, average sieved ash (< 45 μm) compositions from arc volcanoes can be approximated as 33 wt% glass, 50 wt% plagioclase and 10 wt% pyroxenes and for intraplate eruptions as 40 wt% glass, 25 wt% feldspar and 20 wt% pyroxenes. Intraplate samples typically contain 5 wt% more ‘minor’ phases than arc samples.

We highlight the changes in composition for the sieved ash in comparison to the bulk in Fig. 5d, particularly for minor phases. A trend of decreasing glass fraction with smaller particle size is consistent for both tectonic settings. This is more pronounced for intraplate samples, averaging 6 wt% less glass compared to the unsieved ash. Smaller absolute variations in phase fractions are highlighted in the right-hand panel, showing relative changes in phase fraction. Many common changes were seen in both tectonic settings, crystalline silica, salts and ‘other’ phase fractions were higher in the finest ash (< 25 µm), while glass, amphibole, iron oxides, and phosphate fractions were lower. However, in intraplate settings changes were more pronounced for glass, amphibole, and phosphates. Pyroxenes and feldspar show opposing changes at the finest grainsize for arc samples, feldspar and clinopyroxene decrease while orthopyroxene increases, while the opposite variations are noted for intraplate samples. Some of the most significant changes in fine ash are seen for crystalline silica (up by 50–100% in < 25 μm size fraction), salts (up by 40–60%) and iron oxides (down by approx. 50%). Regarding crystalline silica, it is interesting to note that we measured cristobalite exclusively in arc samples, while tridymite and quartz were measured in all tectonic settings. The large apparent change in ‘other’ phases for intraplate samples is affected by very large variations in zeolite contents for the Eyjafjallajökull sample, which is discussed later; however notable increases are found in the ‘other’ category for both arc and intraplate samples.

**Figure 7:**
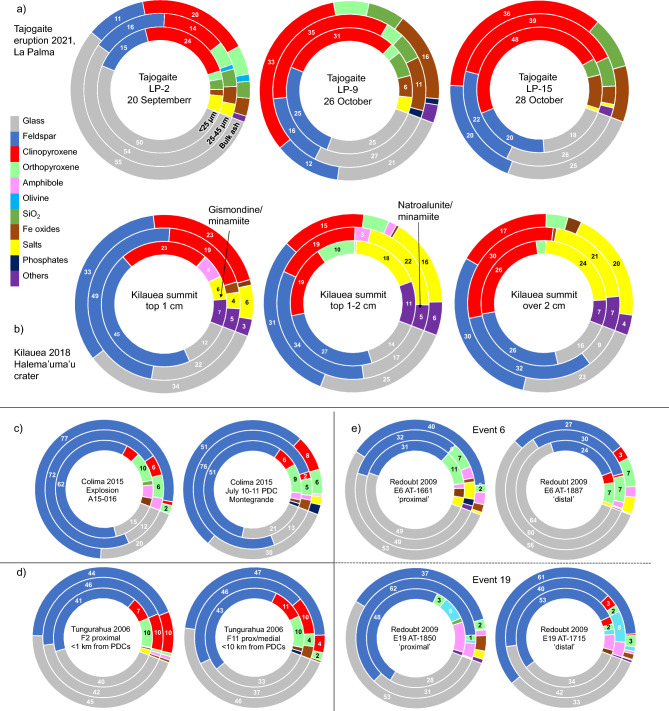
Variations in composition with distance and time. (**a**) Phase fractions for three airfall ash samples from the 2021 Tajogaite eruption on La Palma, Canary Islands, with size fractions nested in the donuts as labeled. Sample LP-2 was collected at the beginning of the eruption, while samples LP-9 and LP-15 were collected approximately one month later. (**b**) K﻿īlauea ash sampled at varying depth from the summit caldera rim following the 2018 eruption. The identity of the most common phases grouped under ‘Other’ shown. (**c**) Volcán de Colima ash from an explosive event (left) and overbank PDC deposits (right), separated by approximately three months in 2015. (**d**) Two samples from the 2006 eruption of Tungurahua. Although the samples were collected at varying distances from the vent, the proximity to major PDCs appears to dominate the measured modal componentry and distance from PDC runout is given. (**e**) Two pairs of samples from the Redoubt 2009 eruption collected at relatively proximal and distal locations for two eruption episodes (Event 6 (E6) and Event 19 (E19)). An increase in glass fraction for the distal samples is clearly seen for Event 6 and for the sieved particles in Event 19.

Changes in phase fractions and size-fractionation are also evident with between different eruption episodes and with transport distance for eruptions with multiple samples. Specific comparisons for several eruptions with multiple samples (Kīlauea, Tajogaite, Colima, Redoubt, and Tungurahua) are shown in Fig. 7, showing variations with pyroclastic density current inputs (Colima and Tungurahua in Fig. 7d), ash cloud transport (Redoubt) and tephra burial depth (Fig. 7b) in the Halema’uma’u crater rim, K﻿īlauea. Very high salt fractions together with alteration minerals (“other”) likely representing decreasing degrees of hydrothermal alteration with depth in the Halema’uma’u crater, a pattern that is stratigraphically inverted in the deposit. For two eruptive events from the 2009 eruption of Redoubt, Alaska, we plotted modal compositions in Fig. 6e. For both Event 6 and Event 19, separated by 12 days^58^, we compare results from relatively proximal and ‘distal’ samples between the bulk and finest (< 25 μm) grainsizes. ‘Distal’ samples show varying change in glass fraction of fine grains, increasing for Event 6 but decreasing for Event 19. The feldspar fraction decreases with distance for both sample sets, while crystalline silica and amphibole increase substantially with distance for Event 19 and Fe-oxide and salts are effectively absent in the distal samples. For samples from two episodes of the 2021 Tajogaite eruption on La Palma, Canary Islands, separated by approximately one month, we plot size-separated modal compositions in Fig. 7a. An > 25% increase in crystallinity is observed; on top of this, more subtle size-dependent variations are observed, for instance the strong depletion in clinopyroxene observed for the 25-45 μm fraction in the 20 September sample is not reproduced in the 26 October sample, while the dramatic size-fractionation of iron oxides is only apparent in the later samples.

**Figure 8:**
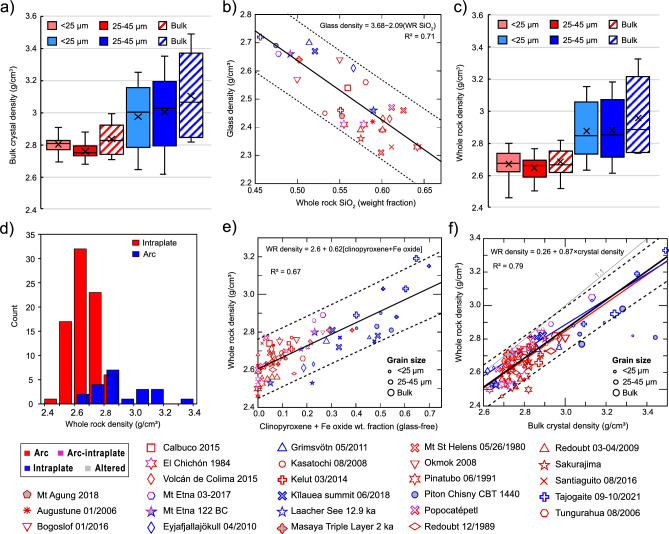
Density, chemistry, and size sensitivity. (**a**) Boxplots show bulk crystal density by size fraction for arc and intraplate samples. (**b**) The calculated glass density is sensitive to WR SiO_2_, as shown with a linear regression (solid black) and 95% confidence intervals for individual points (dashed lines). The regression has a poor fit for arc data alone. (**c**) Boxplots showing whole rock density with grain size; distributions for each tectonic setting are highlighted in (**d**). (**e)** Whole rock density is plotted against primary mineral fractions (e.g., glass- and secondary mineral-free) of clinopyroxene + Fe oxides. Bulk crystal and whole rock density if shown for all samples in (**f**). For panels (**e**) and (**f**), regressions are plotted for all non-altered samples (black, with 95% confidence limits for individual data points in dashed lines and equation given at top of plot). The 1:1 line and regression equation are shown. For panel (**f**) arc (red) and intraplate (blue) linear regressions are also shown.

Measurement of the weight fractions of crystal phases allows the bulk crystal density to be calculated. Crystal densities in the ICDD libraries for the specific mineral composition assigned in MDI Jade were used for most phases, supplemented rarely by values from the Mindat.org website (see Supplementary Table 5). The ‘bulk’ crystal density was calculated from the weighted average of crystal volume fractions. Bulk crystal densities varied between 2.6 and 3.5 g/cm^3^, with mean values of 2.8 ± 0.01 g/cm^3^ for arc and arc-intraplate samples and 3.07 ± 0.04 g/cm^3^ for intraplate samples. Finer particle sizes had the small but repeatable effect of decreasing the bulk crystal density of arc samples, with the lowest value of 2.75 g/cm^3^ for 25-45 μm samples likely caused by the higher feldspar fraction in this size range, up to 2.83 g/cm^3^ for bulk samples; consistent decreases from 3.06 g/cm^3^ for bulk to 3.0 g/cm^3^ for < 25 μm samples were found for intraplate samples (Fig. 8a). Glass densities were modeled from groundmass glass compositions retrieved from the literature for each sample (see Supplementary Table 6): the weight fractions of major element oxides were input to a multicomponent model for room temperature density estimation of glasses^59^. Lower-Si glass compositions (SiO_2_ < 55 wt%) typically fell outside the application limits of the model and were estimated based on a linear regression through 27 output data points spanning 55–80 wt% SiO_2,_ with an R^2^ of 0.92 (Supplementary Fig. 2)_._ Glass densities (retrieved from the modeled and projected results) ranged from 2.31 to 2.72 g/cm^3^, with the phonolitic Laacher See glass showing the greatest deviation from the regression. Modeled glass densities were correlated with WR SiO_2_ and we plot a linear regression of

4 $$Glass\;density = 3.68 - 2.09 \cdot \left( {{\text{WR }} {\text{SiO}}_{2} } \right),$$

with an R^2^ of 0.71 (Fig. 8b).

We calculated the mean ratio between the modelled glass density and bulk crystal density, and found they clustered around 0.873 ± 0.15 for all arc and intraplate samples and size splits, with no significant changes with tectonic setting or size fraction. From the glass density model and XRD results, we calculated the glass volume fraction and used this to calculate a bulk density for each sample (often termed as dense-rock-equivalent (DRE) density, here labelled whole-rock density). Median whole rock density was 2.66 ± 0.01 g/cm^3^ from arc volcanoes and 2.85 ± 0.04 g/cm^3^ from intraplate volcanism (Fig. 8c). These average values are quite consistent for all particle sizes for arc volcanoes; for intraplate samples the unsieved ash showed slightly higher median densities of 2.89 ± 0.06 g/cm^3^. The narrower distribution of density values for arc samples is highlighted in Fig. 8d.

It is clear that the primary crystal assemblage and their density are a key factor in bulk density. We find that the (glass-free) proportion of clinopyroxene + Fe oxides (see Fig. 5 and Eq. 3 for a method of estimation) is correlated with whole-rock density (Fig. 8e), with a linear regression of

5 $$Whole\;rock\;density = 2.6 + 0.62 \cdot \left[ {{clinopyroxene} + {Fe\;oxide}} \right],$$

and R^2^ of 0.67. By considering the bulk crystal density the fit may be improved (Fig. 8f) as

6 $$Whole\;rock\;density = 0.26 + 0.87 \cdot \left[ {crystal\;density} \right],$$

with an R^2^ = 0.79, but this is a harder value to obtain. However, it is clear that bulk crystal density is the dominant control on whole-rock density with the exception of the highly-altered K﻿īlauea samples. The regression provides an estimate within ± 0.15 g/cm^3^ for 95% confidence limits. The regression is more robust for intraplate samples, as arc samples cover a smaller range of bulk crystal density. Further histograms of bulk crystal and glass whole rock, and crystal:glass density ratios are shown in Supplementary Fig. 3.

## Discussion

In this study, we constrain petrographic and geochemical characteristics of volcanic ash in 25-45 μm and < 25 μm size ranges that are most relevant for sustained atmospheric transport^28, 60^ and compare these to as-collected bulk ash samples for two distinct tectonic settings: arc-volcanoes and intraplate volcanoes (with several examples from complex arc locations that include intraplate components defined here as arc-intraplate). Separating samples by tectonic environment is logical from a genetic perspective as this will determine the contrasting major element chemistry of the primary melts, the effects of silica saturation, oxidation state (*f*O_2_), and volatiles on crystallization of phases.

Generally, arc magmas are relatively volatile-rich, oxidized (e.g. average oxygen fugacity of QFM for intraplate volcanoes and QFM + 1 (log units *f*O_2_ referring to the quartz-fayalite-magnetite buffer) for arc volcanoes^48^), and silica-saturated compared to intraplate magma. For relatively oxidized, H_2_O-rich, and silica-saturated melts beneath arc volcanoes^61–63^, the crystallization trend is defined by the calc-alkaline magma series. This entails early crystallization of clinopyroxene and magnetite followed by Ca-rich plagioclase and the replacement of olivine with orthopyroxene leads, which leads to successive enrichment of SiO_2_ and alkali oxides in the remnant melt, eventually producing alkali-rich feldspar and crystalline silica^64, 65^ with high degrees of crystallization (and/or with some level of assimilation^50^).

In the relatively more reduced and H_2_O-limited mantle-derived melts feeding intraplate volcanism, crystallization follows the tholeiitic sequence^62, 66^ where olivine and plagioclase remain stable as crystallization progresses, leading to saturation of Fe–Ti oxides and alkalis. Tholeiitic magmas follow similar early crystallization steps, but more fractionated magmas resemble dacitic-to-rhyolitic calc-alkaline rocks in mineralogy and can be distinguished by Fe- and Ti- enriched phases and bulk chemistry^49, 63^. For silica-undersaturated alkaline series that are also common in intraplate settings, melt evolution leads to later stage crystallization of clinopyroxene, Fe–Ti oxides, biotite, nepheline and (depending on pressure and volatile contents) plagioclase^67^.

As such, a distinction in modal mineralogy between these broad tectonic categories is expected. Our dataset shows clear variations in the fractions of feldspar, clinopyroxene, and glass (Figs. 5–6) which constitute the major phases for these magma series. Feldspar dominates the mineral assemblage for arc magmas, while feldspar and clinopyroxene occur in approximately even fractions for intraplate magma < 50 wt% SiO_2_, but at a 2:1 ratio for samples with 50–55 wt% silica. In general, the mineral fractions diagram shown in the inset of Fig. 4 underestimates the fraction of feldspar and overestimates the clinopyroxene fraction compared to our ash samples. Both orthopyroxene and Fe oxides are not considered in this idealized diagram. Additionally, the mineral fractions diagram overstates amphibole and mica fractions compared to most natural samples, and it will likely depend more on the tectonic setting (and indications from past erupted materials) than silica content. Taken together, although it describes the expected trends in composition for the major crystal phases with varying silica, the diagram is not a reliable indicator for ash composition.

Within the size ranges studied we find some consistent variations with size: in all cases, the glass and iron oxide fractions decrease and crystalline silica and adhering salts increase at fine particle sizes (Fig. 5b–c), consistent with previous studies^31, 68, 69^. The observable average changes with grain size mask greater variations found for some types of eruption. For example, all large VEI 5 + eruptions except Mount St. Helens had substantially higher glass in the < 25 µm ash fraction and the highest feldspar fraction in the bulk. These data, together with grainsize-sensitive trends in space and time (e.g., Figs. 3, 4, 5 and 6 and results of previous studies^29–31^ demonstrate the importance of size-dependent partitioning when considering volcanic ash composition, and thus the need to measure these materials and constrain their composition and variations.

Such partitioning likely has a dependency on the size and material properties of the mineral phase, which are largely determined by the storage and ascent history of the magma. Crystallization of a mantle-derived magma is generally considered to occur in at least two stages: deeper, slower crystallization in magma reservoirs at 10s to 100s km depth, and relatively fast crystallization occurring during transitory ascent, ‘shallow’ storage, and emplacement. To mobilize magma from a reservoir at depth, a minimum melt fraction of 30–50 vol% is generally required^70^. This leads to a characteristic bimodal size distribution of crystals in magma: (i) phenocrysts formed during long-term storage and entrained in ascending magma and (ii) micro-to-nanolites formed during shallow storage and final pre-eruptive ascent. This bimodal distribution is enhanced in arc settings, as crystallization in water-saturated arc magmas during deeper magma ascent may be suppressed, since magma temperatures can exceed the water-saturated liquidus until shallower depths are reached^71, 72^, where rapid crystallization of microlites and nanolites commonly occurs. In all cases, the period at which magma remains at shallow depth before eruption can strongly affect the remaining melt fraction, which is quenched to glass upon eruption. This is also a function of magma ascent rate since rapid ascent reduces the time available for devolatilization- and cooling-driven microlite crystallization. Such rapid ascent is also implied to increase the explosivity of an eruption as decoupling of bubbles and permeable loss of volatiles from the magma is inhibited. Therefore, we might expect to find a decrease in the total crystal fraction with increasing explosivity, a conclusion supported by studies measuring the timescales of ascent via element diffusion profiles in crystals and melt^73, 74^. Mafic melts are typically less likely to erupt explosively due to low viscosities; however, they are further from their liquidus at shallow depths than silica-rich melts and very rapid crystallization during ascent may encourage explosive fragmentation^75^.

### Interpretation of proximal ashfall deposits

Ash samples collected for this study were mainly collected from 10s of kms from the volcanic vent. The characteristics of the samples therefore represent the dynamics of proximal-to-medial fallout (i.e., fallout prior to long-distance atmospheric transport). The volcanic ash in the ash cloud, responsible for the most widespread impact of the ash, are not described by the proximal deposits; indeed, opposite fractionation trends in terms of modal composition and a sharp contrast in grainsize distributions could be expected between proximal deposits and the medial-to-distal ash cloud and deposits^16, 52^. This is exemplified by our observations showing that the finest particles in proximal deposits are initially glass-poor with respect to the bulk (Fig. 5b–c). The implication is that glass-poor fine particles are preferentially deposited before glass-rich particles (due the capacity of the glass phase to host pore space and the relatively low density of the glass phase) leading to the well-documented increase in glass-rich fines with increasing transport distance in the eruption cloud^52^. However, particle aggregation and premature fallout of fine particles^55^ is ubiquitous during ash cloud transport^76^ and may favor glass-rich particles^77^ (perhaps via enhanced salt formation^57^). This prevents a simple relationship between deposited and airborne ash from being articulated and ensures fine ash content even for very proximal deposits. It should be noted that < 4 µm particles sampled directly from volcanic ash clouds have been observed to be crystal-rich compared to the bulk^60^; it is possible that favorable aggregation of fine glassy particles^76, 77^ leaves fine crystalline fragments with the longest atmospheric residence times.

Fractionation of (25-45 μm) and (< 25 μm) particles showed some interesting dependencies on density and size. The highest feldspar fractions were often found in intermediate grainsize samples, both on average (Figs. 4–5) and pronounced for many individual samples (e.g., Figs. 3–4). In contrast, clinopyroxene is depleted in the 25-45 µm size range for both arc and intraplate samples. These patterns are likely to reflect the density contrast between these phases and we could simply infer that denser phases are depleted in both ground deposits and the ash cloud at significantly more proximal locations than feldspar for the same particle size range^52^. However, in the < 25 um size-range, intraplate samples are enriched in clinopyroxene while for arc samples feldspar is depleted. These counterintuitive trends may be explained by a population of feldspar microlites generating free crystals and crystal fragments in the 25-45 µm size range^78^ particularly in arc samples^79^. We infer that the size distribution of major crystal phases plays a key role due to phase-boundary fracture^80^, where larger crystal populations (i.e., microlites-to-phenocrysts) are depleted in fine particles at a faster rate than smaller populations of crystals (i.e., nano-microlites). The effect is greatly enhanced for high-density crystals due to preferential fallout of denser particles within any given size range^31, 52^, as exemplified by the dramatic reduction in iron oxide fractions in fine particles for intraplate volcanism where these phases are present as phenocrysts.

The typical size distribution of crystal phases is in large part controlled by crystallization series and magma ascent history, together with assimilation and/or mixing of the pre-eruptive magma^50^. Thus, while the dominant modal signatures of magmas appear to be established via deeper crystallization of phenocrysts, microlite crystallization can act to enhance the contrast between pyroxene fractions for arc and intraplate magmas. The restricted grainsize resolution in this study opens the possibility that further important partitioning of phases may be found, particularly in particles < 25 μm, a hypothesis which is supported by a small number of available studies^29–31, 60^.

**Figure 9:**
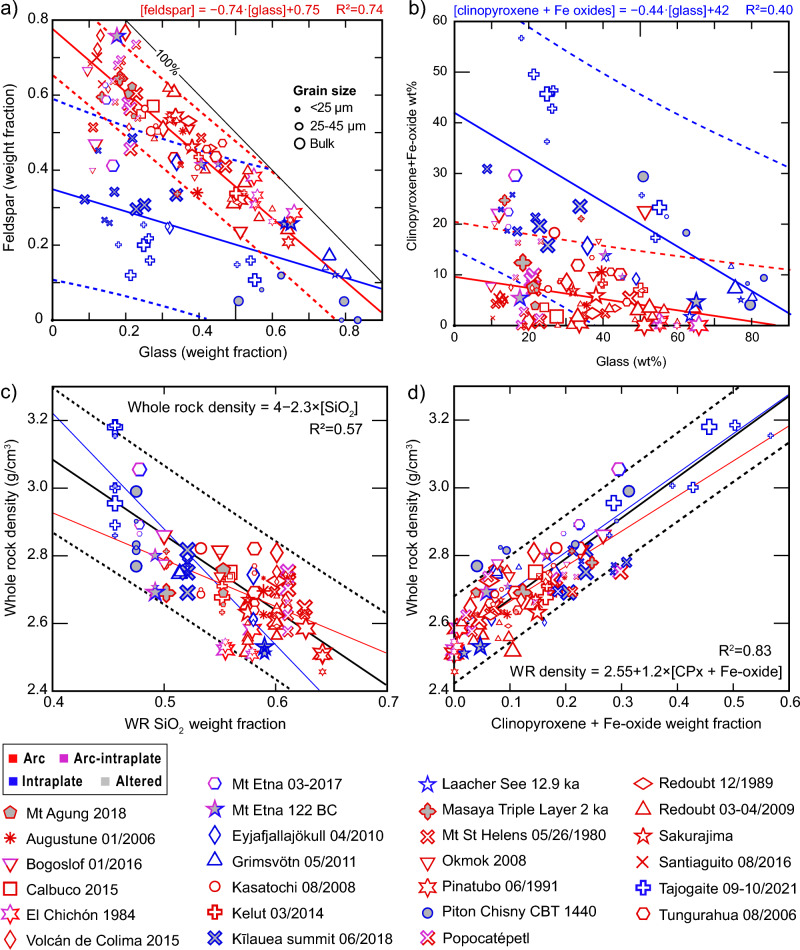
Relation between glass and mineral phases and correlations with whole-rock density. Different trends are seen between intraplate and arc samples for feldspar vs glass fraction (**a**) and clinopyroxene + Fe oxide fractions (**b**). Whole rock density is shown against SiO_2_ fraction (**c**) and against clinopyroxene + Fe-oxide fraction (**d**). In most cases the fraction of dense mineral phases is the dominant control on the bulk density; poorer fits are found with bulk chemistry, with SiO_2_, shown in (**c**), and TiO_2_ showing the best correlations. Symbols match the legend, symbol size indicates particle size fraction as shown in (**a**). Solid lines show linear regressions and dashed lines show 95% confidence intervals for individual data points, colored to match the tectonic setting. Regressions for all data points are shown as black lines, with the equation and coefficient of determination (R^2^) given in each panel. Clinopyroxene is abbreviated to CPx in the regression equation in (**d**). Lines showing 100% fractions for the plotted phases are drawn on panels (**a**) and (**b**). Altered samples (shaded grey) are shown but not included in regressions for all panels.

We investigated the relationship between the major mineral phases and glass fractions by plotting their weight fractions for all samples in Fig. 9. For arc eruptions, the data show that feldspar fractions remain above 25 wt% in all cases and glass + feldspar fractions typically comprise 70–95 wt% and are inversely correlated (Fig. 9a), with linear regression

7 $$\left[ {feldspar} \right] = - 0.74 \cdot \left[ {glass} \right] + 0.75,$$

with an R^2^ = 0.74. Glass-depleted data points that fall off this trend are either associated with high lithic fractions (due the vent widening at Santiaguito and flank collapse at Mount St. Helens), mafic Plinian eruptions (Masaya, Etna 122 BC) and complex (Popocatepétl) or back-arc settings (Bogoslof). Intraplate eruptions show a shallower trend with lower feldspar fractions and a broader spread of data. However, for the relationship between clinopyroxene + Fe-oxides and glass, intraplate samples show a considerably steeper negative correlation than samples from arc volcanoes (Fig. 8b). The reversed pattern of data distribution between Fig. 8a and 8b suggests that it is the replacement of early onset feldspar crystallization (tholeiites) with pyroxene (alkaline and calc-alkaline) that controls the major modal composition of erupted rocks as a function of pressure and/or water^62, 81^. Correlations between these three major phases are further investigated by plotting glass + clinopyroxene against feldspar (Supplementary Fig. 4) and show that these three phases make up 85–100% of all arc samples and most intraplate samples. Regressions for intraplate and arc settings are similar, with arc samples offset to higher feldspar. Laacher See and K﻿īlauea summit samples fall below the trend observed in Fig. 8c due to substantial alteration; We recalculated glass, pyroxene and feldspar fractions in Eyjafjallajökull samples fall to account for the incorporation of substantial non-juvenile zeolites during the explosive excavation of older, altered units around the summit vent^82^. The lower right corner of both plots in Fig. 8a–b is dominated by highly explosive mafic eruptions; however, where high-silica arc magmas are under-represented in our dataset, highly explosive intraplate magmas are perhaps overrepresented. High-Si arc magmas are typically highly glassy; therefore, we expect the data points to converge in this region with better coverage of high-Si compositions.

Phase concentrations within an ash deposit are expected to vary with particle size and transport distance, since the physics of ash fallout separate denser and less aerodynamic particles closer to the eruption source^52, 54^. However, these dynamics are overprinted by the effects of aggregation-driven fallout, which can dominate proximal ash settling under certain conditions^83^ and simultaneously promote premature removal of fine particles and delayed fallout of coarse particles^55, 84^. Nevertheless, in many cases particle aerodynamics (controlled by size, shape and density) are the primary control on total atmospheric residence time and transport distance^52, 53^. Within our results, whole rock density covers a considerable range for both tectonic categories despite a clear offset to higher densities for intraplate ocean island samples. To investigate possible correlation with chemistry or modal composition, we plot whole rock density against silica content (Fig. 9c) and against clinopyroxene + Fe-oxide weight fractions (Fig. 9d). The linear regression for whole rock density vs silica through all arc and intraplate data points show a linear regression with equation

8 $$Whole\;rock\;density = 4 - 2.3 \cdot \left( {{\text{WR}} {\text{SiO}}_{2} } \right),$$

with an R^2^ of 0.57. This correlation is partly dependent on the magmatic series since mafic oxide concentrations are somewhat codependent with silica due to similar crystallization sequences. However, considerable variability is noted within the calc-alkaline arc dataset the regression through arc data points is particularly poor. Whole-rock density shows a better correlation with clinopyroxene fraction alone (with R^2^ = 0.77), partly due to the relatively high density and fraction of clinopyroxene in intraplate and mafic magmas, but perhaps also related to correlations with melt chemistry (especially Fe, see glass compositions in Supplementary Table 6) and mafic mineral fraction for intermediate to silicic magmas. This is borne out by the regression for clinopyroxene + Fe-oxide fraction against whole rock chemistry illustrated in Fig. 8d, showing a relatively good spread in values for arc samples and a linear regression of

9 $$Whole\;rock\;density = 2.55 + 1.2 \cdot \left[ {{clinopyroxene} + {Fe\text{ }oxide}} \right],$$

with an R^2^ of 0.83.

### Implications for environmental impacts and ash transport modeling

The modal composition and density of volcanic ash are important constraints for a wide variety of disciplines. For example, the bulk optical properties of volcanic ash clouds may be constrained from the weighted optical properties of the constituent phases^85, 86^ and the refractive indices of glasses are correlated with their density^87^. In addition, the specific properties of separate phases (for example their electrostatic properties, optical properties, toxicity, dissolution and weathering rates, oxidation potential, and diffusion rates) are much better constrained than the properties of volcanic ash in bulk. Therefore, better constraints on the modal composition of volcanic ash may benefit modeling and forecasting the diverse impacts of volcanic eruptions across Earth and planetary science, environmental science, respiratory health, and engineering.

Glass fractions are perhaps the most important for a spectrum of environmental interactions. Glass is usually the main phase involved in fixing volatiles from the eruptive plume^15^ due to its relatively high elemental diffusivity, a process that encourages particles to form aggregates^57, 88^, which is often an important proximal process in removing fine material and volatiles from the ash cloud and transporting coarse particles to greater distance^55^. An important observation of our work for environmental impact is that the fraction of salts is higher for < 25 μm particles compared to the bulk. Given that glass fractions are relatively depleted in fine particles from the proximal location in our study, it should be expected that the salt fraction is substantially higher in the glass-rich fines entrained in the ash plume at medial-distal locations. Therefore, fine ash may be an important host for S- and Cl- bearing aerosols in the atmosphere following large volcanic eruptions. Salts can significantly change the reactivity of the particles, but also affect the bulk density and optical properties of the particles^57^. The effect of salt crystallization on iron mobility due to oxidation of iron in the glass^89^ potentially increases the environmental impact of far-traveled ash, despite the rapidly decreasing fraction of iron oxides in finer particles.

Finally, we emphasize two topics for further research. The grainsize-dependent phase partitioning that we present for the first time across a global ash sample set are dependent on tectonic setting and eruption magnitude; however, our results suggest that further important partitioning may occur at finer grainsize ranges. These fine grainsizes (e.g., PM10, PM2.5) are particularly important for ash resuspension^19, 90^, distal atmospheric transport^55, 91, 92^, aerosol interaction^15, 69^ and for respiratory health^93, 94^. Future research into the modal composition and chemistry of PM10 to nanoscale ash will be critical in completing our understanding of volcanic ash and integrating understanding and terminology between atmospheric science and volcanology.

Our results also showed significant increases in glass and decrease in pyroxenes for relatively distal vs relatively proximal ash samples (Fig. 7d–e). These transport-related trends are likely due to aerodynamic factors driven by size, shape, and density^55^ and suggest that transport-related sorting may become an increasingly important process in determining the composition of ash clouds as they age. We note the scarcity of atmospheric sampling of volcanic ash clouds in comparison to equivalent fallout samples. Future sampling campaigns aimed at airborne ash from varying distance and height in volcanic ash clouds would be of great benefit in determining the evolution of ash cloud composition during ash dispersion, a crucial question for ash detection and hazards from airborne and fallout.

## Materials and methods

### Materials

The ash samples were collected and provided for this study by colleagues across the globe (Fig. 1), with a notable set from the Alaskan Volcano Observatory (Supplementary Table 1). In total, data were collected on 40 ash samples from 28 eruptions with bulk chemistry ranging from basaltic to dacitic and basanitic to phonolitic (Fig. 1b). Of these, 23 samples were collected from eruptions in the past 50 years. Details and appropriate references for further information on each sample can be found in Supplementary Table 1. We present literature values for the whole-rock (e.g., bulk) chemistry for each sample in Supplementary Table 2, and plot total alkalis vs silica in Fig. 1b.

For arc volcanism, we present data from 16 eruptions from island arcs (e.g., the Aleutians) and continental arcs (e.g., Andes) across a broad range of composition and eruption magnitude, including the Plinian eruptions of Pinatubo and Masaya, and recent eruptions of Kelut, Calbuco, and Colima. Relatively proximal and ‘distal’ sample pairs from the same eruptive event were received for the Redoubt 2009 eruption, the Tungurahua 2006 eruption and the Calbuco 2015 eruption. In the latter case, only the relatively distal sample was included in the study as the proximal sample was lapilli-rich and contained insufficient fine material or XRD measurements. Samples from arc volcanism are dominantly eruption-plume-derived ash, although contributions from pyroclastic density currents (PDCs), which are relatively enriched in fine particles and glass^20, 95^ are important for the Tungurahua samples, which contain a significant fraction of co-PDC ash^96^ and the Colima CMG003 sample^97^, which was taken from a co-PDC overbank deposit adjacent to the flow channel. The Mt Agung sample was collected from within an abandoned structure; the deposit showed precipitation of sulfuric minerals and was saturated with water. The Masaya Triple Layer sample (150 BC eruption) was collected from a weathered exposure 3.5 km from the vent. Both these samples were classified as ‘altered’ and symbols are shaded grey; all altered samples were excluded from regression calculations in all cases.

For intraplate volcanism, all samples are from ocean islands (K﻿īlauea, La Palma (Tajogaite), Réunion, and Iceland (Eyjafjallajökull and Grimsvötn)) except Laacher See, which is fed by the subcontinental Eifel mantle anomaly^98^. Intraplate ash samples are derived from the eruption plume during mixed effusive-explosive activity (e.g., K﻿īlauea, Tajogaite) or dominantly explosive activity (Grimsvötn, Eyjafjallajökull). The Eyjafjallajökull samples was collected during the early eruption phase that may have involved phreatomagmatic fragmentation due to glacial melt input^99^. The Tajogaite samples from October 2021 were collected by the authors directly from fallout at locations west of the vent; the LP-2 sample was collected at the airport, at a similar distance E of the vent prior to rainfall. At K﻿īlauea, samples were collected from the crater rim, from deposits of intermittent explosive activity during subsidence of the Halema’uma’u crater. These samples contain cream-colored secondary surface salts and are considered to be altered either on the Halema’uma’u crater floor or following deposition on the crater rim. For the Laacher See sample, fine ash was produced by experimental abrasion of pumice clasts^78^ and therefore better represents co-PDC ash than fallout deposits; a significant contribution from ash produced in PDCs has been shown for the Laacher See 12.9 ka eruption^100^. The Réunion samples were collected from the base and top of an exposure of the AD 1440 Chisny Black Tephra deposits^101^. Both the Laacher See and Chisny deposits have weathered and are considered to be altered.

A small subset of volcanoes occur in more complex settings: At Popocatépetl, contributions from asthenospheric mantle are suspected to generate unusual OIB-like magma, resulting in magmas that record subduction and intraplate signatures^102^. Nearby, El Chichón is set back from the main arc front and is located above a subducted transform ridge. The unusual K-rich magmas have been attributed to an upwelling from underlying asthenospheric magma via slab tear(s)^103^ and deep serpentinization of the ridge basalts^104^. Bogoslof, in the Aleutian arc, is a back-arc volcano with magma compositions enriched in alkalis. The Bogoslof sample is likely to derive from eruptions modified by ingress of seawater into the eruption vent, generating Surtseyan-style activity. The sample was collected at Dutch Harbor, 90 km from the vent, representing one of the farthest-traveled samples in the data set. In addition, we present data from Plinian (122 BC) and recent (2017) eruptions at Mt. Etna, which is likely fed from combined intraplate and subduction sources, with post 1970 eruptions bearing a stronger (yet still minor) subduction-related chemical signature^105^. The Plinian sample is considered altered due to weathering. For simplicity, we group samples from these complex volcanoes as ‘arc-intraplate’, marked by pink symbols in Fig. 1 and other figures.

Characterizing the modal compositions of volcanic ash in nature presents a particular set of challenges due to poorly resolved partitioning of phases with grain size. This is affected by fragmentation mode, in addition to transport, aggregation and fallout related sorting of the volcanic ash cloud^29, 52, 53, 95, 96^. Therefore, composition is sensitive to sampling location and partitions between airborne and fallout ash. All samples included in this study were collected from fallout deposits less than 100 km from the source vent. Although these proximal samples do not represent long-lived fine ash that has the widest dispersal, airborne and/or distal volcanic ash samples have not been commonly collected and a representative dataset cannot be compiled. With this caveat, the ubiquitous proximal sampling allows internally consistent comparisons to be made between samples in the dataset.

## Methods

Sample handling and laboratory size separation methods may significantly affect the measured composition of fine ash samples^30^ and the detection of secondary surface phases. All samples were gently dry-sieved by hand to three size ranges: > 45 μm, 25-45 μm and < 25 μm. Sieving below 63 µm has been considered impractical by many researchers, however this is likely to relate to mechanical stack sieving using a shaker rather than to hand-sieving. Hand-sieving down to 32 µm has been used for 300 volcanic ash samples^20^ and more recently, hand sieving was employed down to 45 µm^106^; in both case hand-sieving was chosen in order to mitigate abrasion during sieving. We took the following steps to maximize sieving efficiency and reproducibility:

- Drying at 85 °C for 1 h or more prior to sieving.
- Presieving of > 125 µm fraction.
- Hand sieving for short durations (~ 2 min) with a tightly-fitting lid.
- Sieving of small sample volumes (not more than 15 g per sieve run, less for finest sieve).
- Separate and successive sieving of < 45 and < 25 µm fractions (i.e., no stacked sieves).
- Tapping on lab countertop 2–3 times during hand sieving to prevent blinding.

The ability to effectively deblind sieves by tapping and employ a wave-like motion during hand sieving has provided us with effective (and reasonably efficient, although multiple sieve runs are often required to obtain sufficient particles in the < 25 micron range) method to separate < 63 micron size fractions.

We used a Spex SamplePrep 8000M alumina ball grinder to dry-mill bulk (unsieved) samples for up to 25 min prior to XRD analyses. Sieved particles were measured directly, without further milling. X-ray diffraction (XRD) measurements were made using a Bruker D8-ECO Powder X-ray Diffractometer in the Cornell Center for Materials Research (CCMR). The instrument has a Cu K-alpha X-ray source excited by a 40 kV, 25 mA electron gun and a silicon strip detector. Measurements were made at angles of 10–80° for most fresh samples and 5–65° for weathered samples, with an increment of 0.0195° and a dwell time of 0.4 s. To filter Fe fluorescence, we increased the lower discriminator on the detector from 0.110 to 0.182 V, as determined experimentally by CCMR technicians. Depending on sample volume, powder samples were held in either 5 or 10 mm diameter wells in single-crystal quartz sample holders. Comparison between results of 5 and 10 mm sample holders show a good degree of similarity. Spectra were analyzed in MDI Jade 10 software using the ICSD database of crystallography data. We used the Degree of Crystallinity approach to quantify the glass content of the ash samples^107^, where the area under amorphous peaks is divided by the area under crystalline plus amorphous peaks. Peak fitting was conducted semi-automatically for crystalline peaks after applying a baseline following the amorphous peak; crystalline peaks were carefully picked by hand and then fit using the ‘Profile fitting’ algorithm in MDI Jade. Amorphous peaks were picked after applying a linear baseline and fit by hand. Occasionally, two overlapping amorphous profiles were required to fit the amorphous peak in the spectra. Whole pattern fitting (WPF) with Rietveld refinement was used to quantify phases. The full protocol used for analysis of output spectra is laid out in Supplementary Fig. 1. Accuracy was determined using binary mixtures of San Carlos olivine and pure quartz measured using a 0.1 mg resolution scale. Error was typically < 5%, however the 10 wt% olivine + 90 wt% quartz mixture gave relative uncertainty for olivine between 10 and 20% (1–2 wt%). Absolute error did not exceed 2.5 wt% in all cases (Supplementary Table 3). Precision was monitored via repeated measurements of the Eyjafjallajökull 25-45 μm sample with spectral similarity of 98%.

Output mineral fractions were recalculated to include the glass fraction, which was determined from the ratio of the area under the amorphous peak to the sum area under crystal peaks plus the amorphous peak. To consistently report the output data, specific species of minerals were clustered under broader categories, namely: feldspars, clinopyroxenes, orthopyroxenes, amphiboles, olivines, SiO_2_-polymorphs, iron oxides, phosphates, and salts. Any other species were classified under “other” and included alteration products, sulfides, and phyllosilicates detailed in the “Other ID” and “Notes” columns in the Supplementary Table 4. A list of these minerals, their classification and basic properties is included as Supplementary Table 5. Finally, samples sieved to > 45 μm and < 25 μm were mounted and then imaged using a TESCAN Mira FE-SEM with either backscattered electron (BSE) or energy-dispersive spectroscopy detectors to examine microstructure and check phases assigned by XRD (see Fig. 2).

### Supplementary Information

Supplementary Information 1.

Supplementary Information 2.

## Data Availability

All data generated in this study are included in the figures and supplementary information.
