# Supplementary material for: Phases in fine volcanic ash
Source: Sci Rep. 2023 Sep 21;13:15728. doi: 10.1038/s41598-023-41412-x (PMC10514198; doi:10.1038/s41598-023-41412-x)
Supplement: Supplementary file 1 — Supplementary Information 1. [file 41598_2023_41412_MOESM1_ESM.pdf]

Supplementary information for

Phases in fine volcanic ash

Adrian Hornby, Esteban Gazel, Claire Bush, Kyle Dayton, Natalie Mahowald

Earth and Atmospheric Sciences, Cornell University, Ithaca NY

*Adrian Hornby and Esteban Gazel.

Email: ahornby@cornell.edu and egazel@cornell.edu

Supplementary figures and legends

SI figure 1. XRD analysis workflow

SI figure 2. Glass density model outputs and regression

SI figure 3. Histograms of density outputs

SI figure 4. Glass vs clinopyroxene scatter plot

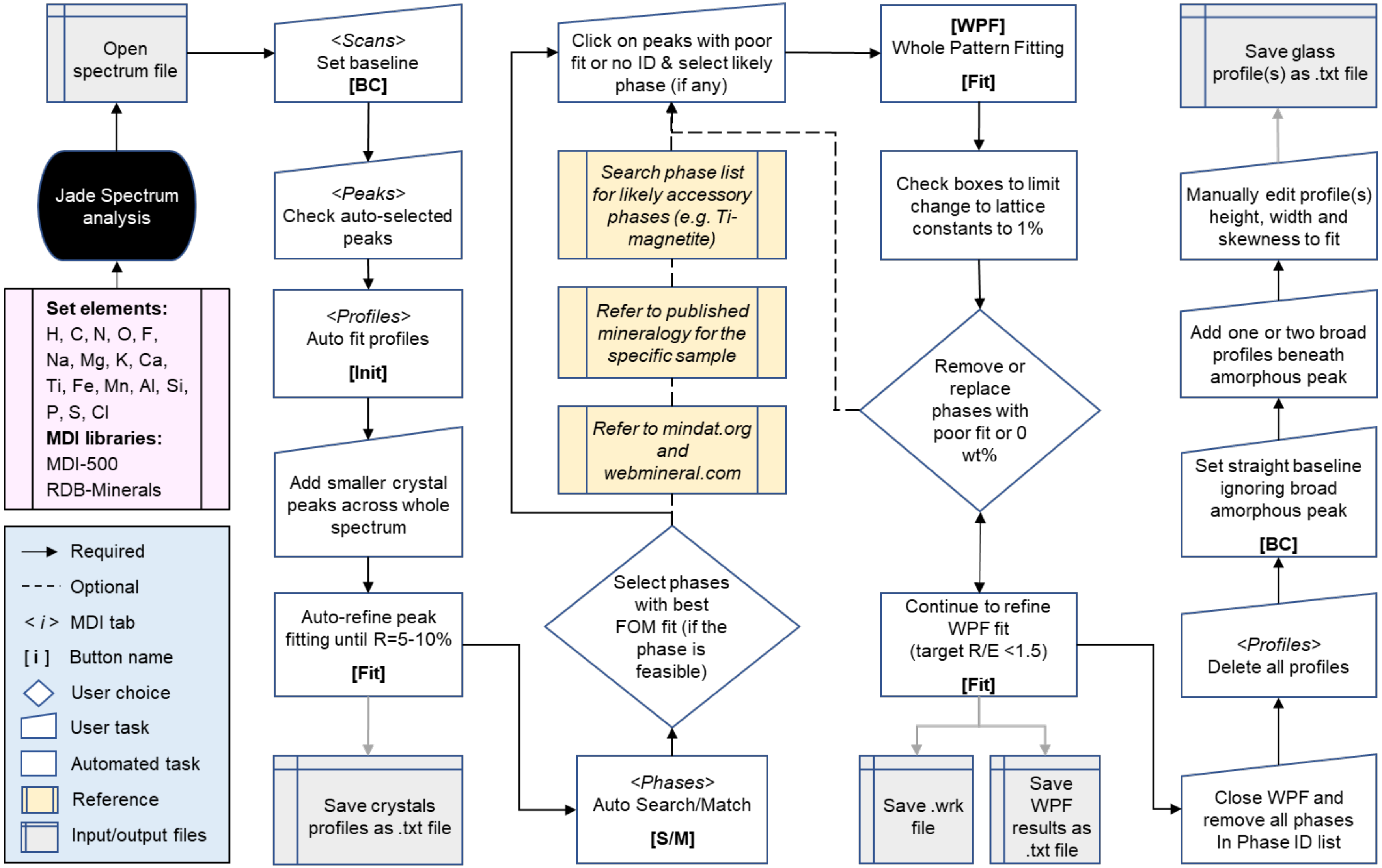

Supplementary Figure 1. Flowchart describing the analysis routine for X-ray Diffraction (XRD) spectra in the software Jade. Box colors and shapes indicate different processes, as described in the key.

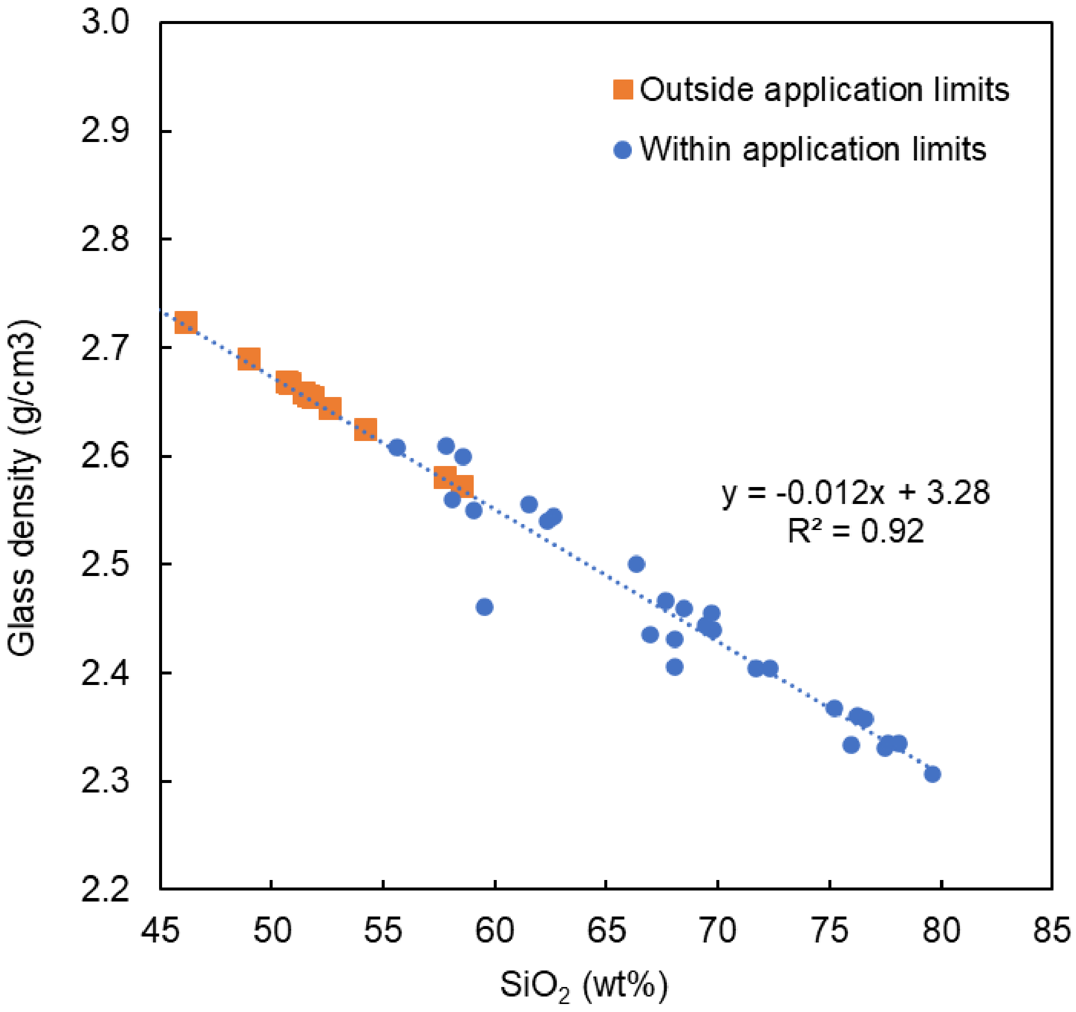

Supplementary Figure 2. Results from the Fleugel 2007 model for room-temperature glass density based on major element chemistry. Blue data points are direct outputs of the model. Mafic (Fe, Mg, Ti, rich) glass compositions the fall outside the model limits are plotted as orange squares. We approximate the density of mafic glasses in our dataset by extending the linear regression (details shown on the figure) through the modeled data to lower silica and/or more mafic glass compositions. These data should obviously be treated with caution, and based on the variance in the modeled data may lead to error of up to 0.1 g/cm^3 in the calculated bulk densities of our mafic samples. The model is available from https://www.glassproperties.com/density/room-temperature/

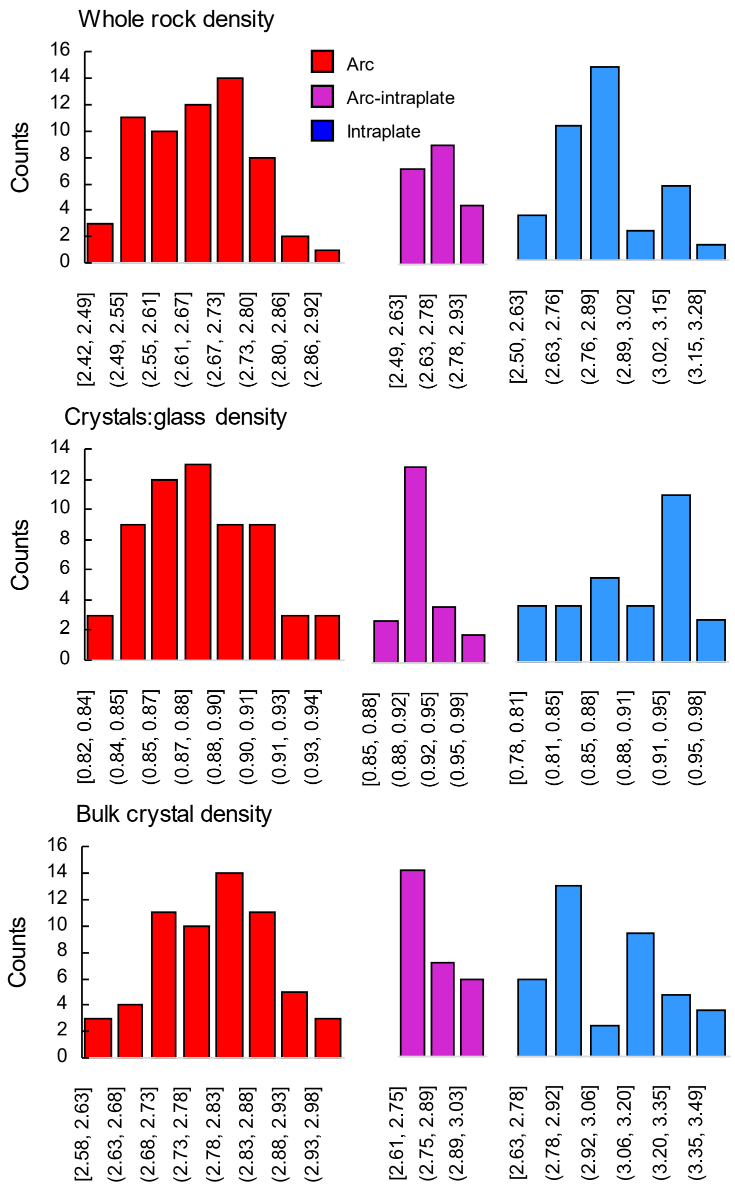

Supplementary Figure 3. Histograms showing the distribution of calculated glass and bulk crystal density data. Colours represent arc (red) intraplate (blue) and mixed arc/intraplate settings (purple).

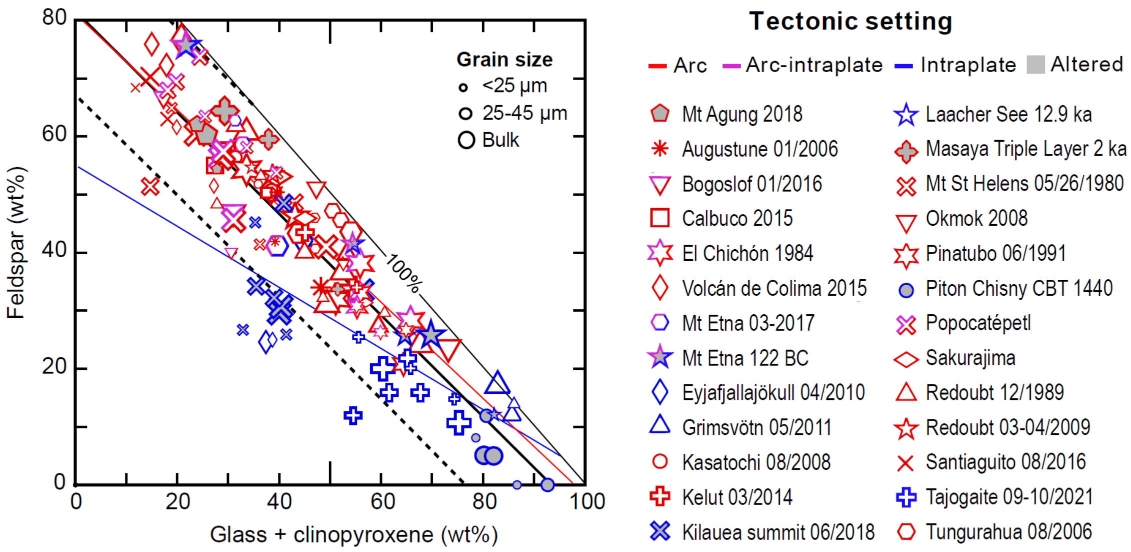

Supplementary Figure 4. Glass vs clinopyroxene fraction for all samples. Regressions are plotted for intraplate samples (blue symbols) and arc + arc/intraplate samples (red and pink symbols).
